# Robust Beamforming Design for Secure V2X Downlink System with Wireless Information and Power Transfer under a Nonlinear Energy Harvesting Model

**DOI:** 10.3390/s18103294

**Published:** 2018-09-30

**Authors:** Shidang Li, Chunguo Li, Weiqiang Tan, Baofeng Ji, Luxi Yang

**Affiliations:** 1 School of Physics and Electronic Engineering, Jiangsu Normal University, Xuzhou 221116, China; 2 National Communications Research Laboratory, Southeast University, Nanjing 210096, China; chunguoli@seu.edu.cn (C.L.); lxyang@seu.edu.cn (L.Y.); 3 School of Computer Science and Educational Software, Guangzhou University, Guangzhou 510006, China; 4 School of Information Engineering, Henan University of Science and Technology, Luoyang 471023, China; baofengji@seu.edu.cn

**Keywords:** SWIPT, nonlinear energy harvesting model, secure vehicular communication, semidefinite relaxation

## Abstract

Vehicle to everything (V2X) has been deemed a promising technology due to its potential to achieve traffic safety and efficiency. This paper considers a V2X downlink system with a simultaneous wireless information and power transfer (SWIPT) system where the base station not only conveys data and energy to two types of wireless vehicular receivers, such as one hybrid power-splitting vehicular receiver, and multiple energy vehicular receivers, but also prevents information from being intercepted by the potential eavesdroppers (idle energy vehicular receivers). Both the base station and the energy vehicular receivers are equipped with multiple antennas, whereas the information vehicular receiver is equipped with a single antenna. In particular, the imperfect channel state information (CSI) and the practical nonlinear energy harvesting (EH) model are taken into account. The non-convex optimization problem is formulated to maximize the minimum harvested energy power among the energy vehicular receivers satisfying the lowest harvested energy power threshold at the information vehicular receiver and secure vehicular communication requirements. In light of the intractability of the optimization problem, the semidefinite relaxation (SDR) technique and variable substitutions are applied, and the optimal solution is proven to be tight. A number of results demonstrate that the proposed robust secure beamforming scheme has better performance than other schemes.

## 1. Introduction

In recent years, vehicular communication has become a very important subject of study among researchers, due to its potential to increase road-safety and reduce traffic congestion [1,2,3,4,5,6]. There has been a tremendous amount of effort and investment from the government and private organizations to develop a means for highly efficient communication. To support various emerging applications, “cellular-connected vehicles” have been recognized as promising new solutions, by integrating vehicles into the cellular network as the new ground user equipment served by the base stations [7,8,9]. Thanks to the superior performance of the Long Term Evolution and the fifth-generation cellular networks, cellular-connected vehicles are expected to obtain significant performance enhancement in terms of all of throughput, coverage and reliability. In fact, the 3rd Generation Partnership Project (3GPP) standardization body released a dedicated set of criteria for providing V2X applications in future cellular networks in 2016 [10,11,12]. Preliminary field trials have also demonstrated that it is feasible to support the basic communication requirements for V2X with LTE networks [13,14,15,16,17].

The key issue with cellular-connected vehicle networks is how to prolong their lifetime. As we all know, the majority of vehicles in V2X networks are sensors that are typically powered by batteries with limited energy, resulting in a constrained network life time [18,19,20,21]. Meanwhile, the development of battery energy storage technology is unable to keep pace with the significant growth of energy requirements. Though replacement of batteries can extend their lifetime effectively, it will bring higher costs and is sometimes impossible. Traditionally, the energy collecting unit could harvest plentiful renewable energy from natural resources such as the hydro energy, tide and wind, etc. [22,23]. However, renewable sources are liable for the limitation caused by an unpredictable climate and change of position. Moreover, it may not be applicable for some application scenarios while harvesting energy from the ambient radio frequency (RF) signals can be controllable. The SWIPT technique exploits different aspects of RF signals, which are signal transmission and power transfer. Therefore, SWIPT technology has attracted great interest from investigators in recent years [24,25,26,27,28]. Utilizing this technology, the battery-powered vehicular communication devices can collect energy from the RF radios to prolong their network lifetime. For future vehicles with a mass of on-board sensors, the SWIPT technique is more meaningful for continuable communications of these on-board sensors, whose power can be provided from base stations or other vehicles with excess power or grid-connected roadside electrical units. Particularly, the authors in [27] designed the optimal beamforming to balance the information and energy transmission. In [28], the harvested energy and the secrecy channel capacity trade-off problem between the information receiver and energy receivers are considered.

However, all of the above works assume that the perfect knowledge of the receivers’ CSI is available at the base station. In practice, the CSI obtained by the base station may be imperfect. There are many factors resulting in imperfect CSI, such as the quantization error, the delay error and the limited capacity of feedback channel. Moreover, as is known to all, the performance of the approaches with perfect CSI may become poor due to the fact that these methods may be quite sensitive to channel uncertainties. Supposing imperfect CSI at the base station, there are several valuable works on robust beamforming design for multiuser SWIPT systems [29,30,31,32,33]. In [29,30], the authors proposed a secure beamforming optimization algorithm for SWIPT systems, where cooperation jamming and the external helper is considered. Khandaker et al. in [31] investigated the chanced constrained beamformer design for a more general SWIPT system in the presence of multiple Eves equipped with multiple antennas. The authors in [32] considered the resource allocation problem for the secure SWIPT multicasting scenario with channel uncertainties, where a low complexity optimization algorithm was proposed. Moreover, the authors of [33] also consider the SWIPT multicasting scenario and address the secure beamforming design problem.

From the above discussion, it is worth noting that most of the existing works focused on the beamforming design supporting SWIPT under the linear EH model, where it is assumed that the collected energy power could be linearly increased by growing the input power of the received RF signals. However, the practical power conversion circuits usually perform a nonlinear feature rather than the linear one due to their nonlinear elements such as two-electrode valves or two-electrode valves connected to the transistor. Therefore, the authors of [34] proposed a nonlinear EH model by fitting over real sampled data based on a logistic function. Then, the resource allocation optimization algorithms for SWIPT and wireless powered communication networks have been attracting increasing interest (see [35,36,37,38,39,40,41]). In [35], authors addressed the max-min signal-interference-noise-ratio (SINR) beamforming design optimization problem in a V2X-SWIPT environment with imperfectly estimated channels at the base station, where a practical nonlinear EH model is considered. In [36,37], the authors discuss the robust transmit beamformer design, joint user scheduling and power allocation for multiple user SWIPT scenario under nonlinear EH model. In [38], the authors designed the beamformer for energy efficiency maximization in a secure SWIPT system taking into account the influence of the nonlinear EH circuits. Then, the authors in [39] investigate the joint optimization of transmit precoding and power splitting ratios in MISO SWIPT multicasting networks with the objective of maximizing the minimum collected energy among all receivers under a generic (including nonlinear) EH model. The multiuser MIMO SWIPT system is also considered in [40,41], where the authors study the transmit beamforming design to maximize the harvested power based on perfect CSI under a generic EH model. The results obtained in [35,36,37,38,39,40,41] demonstrated that a favorable performance gain could be achieved if the beamforming design takes into account the effect of the nonlinear EH circuit instead of a linear EH circuit owing to the mismatch between the linear EH model and the nonlinear EH model. To the best of our knowledge, most of the above works on SWIPT either focus on the optimization of secrecy capacity of information receiver or the transmit power of the base station. It is usually assumed that the CSIs are perfectly available at the base station or the receivers are equipped with one antenna. These motivate us to research uncommonly considered optimization problem of fair power harvesting among all energy vehicular receivers that are equipped with multiple antennas. In addition, the CSIs of base stations to energy vehicular receivers are imperfect.

In this paper, we focus on the optimal secure beamformer design for V2X-SWIPT in a multiple vehicles MISO communication network with multiple multi-antenna energy vehicular receivers, where the nonlinear EH model proposed in [35] is utilized. The CSIs between the base station and the energy vehicular receivers are supposed to be imperfect. Moreover, the artificial noise-aided signal strategy and the power splitting receivers are taken into account. We aim for the design of a beamforming design algorithm maximizing the minimum harvested energy among multiple multi-antenna energy vehicular receivers for the case when the information vehicular receiver is able to collect energy from radio frequency signals. Our design advocates for the dual use of both energy signal and artificial noise in facilitating efficient wireless energy harvest and providing secure communication. The beamforming design is formulated as a non-convex optimization problem. For obtaining a tractable solution, we reformulated the considered optimization problem by applying a S-procedure and variable substitutions with a tractable solution. The resulting reexpressed non-convex optimization problem can be solved via a semi-definite programming based beamformer design algorithm. Furthermore, the obtained solution is proved to be the optimal solution.

The main contributions of this work are summarized as follows:

Firstly, to explore the network performance, an optimization problem is formulated to maximize the minimum harvested energy power among all the energy vehicular receivers, which is subjected to the required SINR constraints at an information vehicular receiver and energy vehicular receivers, the collected energy power constraints at the information vehicular receiver, and the transmit power constraint at the base station.

Secondly, since the considered problem is non-convex and challenging because there are infinite inequality constraints taking into account the nonlinear EH model and coupling among optimized variables. In order to solve the problem, a beamforming design algorithm is proposed based on the S-procedure and semidefinite relaxation (SDR). Moreover, It is proved that the optimal solution can be guaranteed by applying our approach.

Thirdly, our simulation results demonstrate that the proposed secure beamforming design scheme under the nonlinear EH model with channel uncertainties can provide performance gains compared with that under the linear EH model with channel uncertainties. Moveover, it is shown that a trade-off is found between the SINR of the information vehicular receiver and the energy harvested by energy vehicular receivers.

This rest of this paper is organized as follows. The system model and problem formulation is described in Section 2. Section 3 presents a solution for modelling max-min harvested energy power among all the energy vehicular receivers with imperfect CSI and a nonlinear EH model. Simulation results are provided in Section 4 to verify the effectiveness of the proposed robust secure beamforming design algorithm. Finally, we conclude the paper in Section 5.

## 2. System Model and Problem Formulation

**Figure 1:**
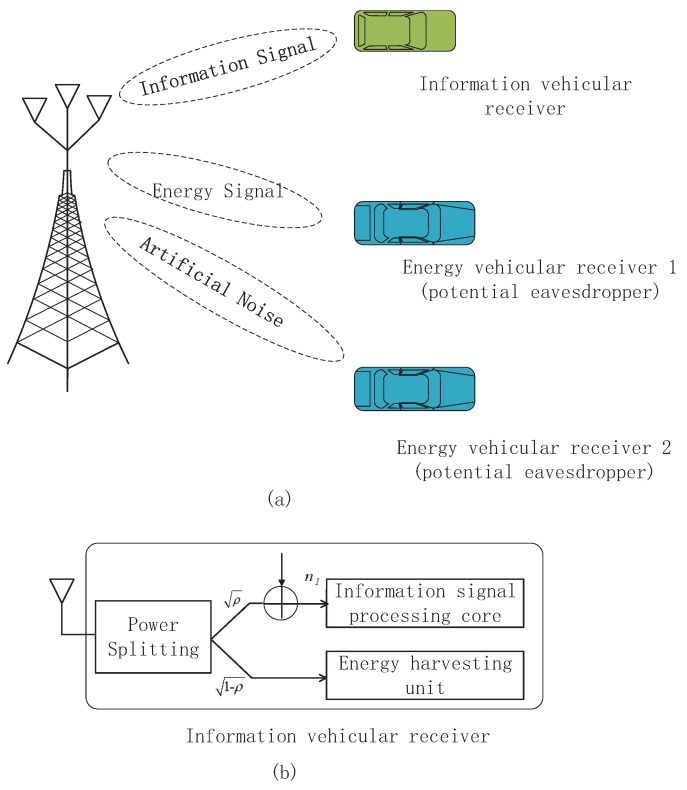
(**a**) V2X-SWIPT system model for two energy vehicular receivers and one information vehicular receiver; (**b**) the block diagram of the information vehicular receiver model for wireless information and power transfer.

We consider the downlink of a V2X-SWIPT communication system that consists of a base station and two kinds of legitimate vehicular receivers, namely one information vehicular receiver and $K_{E}$ idle energy vehicular receivers. The base station is equipped with a $N_{T}$ transmit antenna while the information vehicular receiver is a single antenna device and is able to decode information and collect energy from radio signals, cf. Figure 1. In addition, the $K_{E}$ idle energy vehicular receivers equipped with $N_{R}$ antennae are supposed to collect energy from the radio frequency as they are inactive. The energy vehicular receivers can work in double functions of EH and information decoding. In this scenario, the idle energy vehicular receivers, which cause no interference to the communication channel, can potentially eavesdrop on the confidential information of the information vehicular receiver by switching its working pattern to information decoding since all the legitimate vehicular receivers are within service coverage. Therefore, the idle energy vehicular receivers are potential eavesdroppers that can take care of supplying physical layer security. The channel vector between the base station and the information vehicular receiver is denoted as $h\in C^{N_{T}\times 1}$ and the channel matrix between the base station and the *k*-th energy vehicular receiver is denoted as $G_{k}\in C^{N_{T}\times N_{R}}$, $\forall k\in \left\{1,2,\cdots ,K_{E}\right\}$. In our considered system model, we suppose that the vehicle speed is low. Several simulation and theoretical investigations have shown that V2X communication channels such as h and $G_{k}$ can be modelled as Rayleigh channel fading [42,43,44,45]. It is assumed that our considered system works in a Time Division Duplex (TDD) mode with slowly time-varying communication channels. Hence, the base-station-to-legitimate-vehicular-receiver fading gains, h and $G_{k}$, could be reliably estimated at the base station at the start of each scheduling slot with negligible channel estimation errors. During the data transmission, the information vehicular receiver sends acknowledgement packets to tell the base station of successful reception of data packets. Hence, in this paper, the channel uncertainties for the base-station-to-energy-vehicular-receiver are considered while the CSI of the base-station-to-information-vehicular-receiver is perfectly known at the base station. We model the CSI of the link between the base station and the *k*-th energy vehicular receiver as:

(1) $$G_{k}={\bar{G}}_{k}+\Delta G_{k},\forall k\in \left\{1,2,\cdots ,K_{E}\right\},$$

(2) $$\Xi_{k}≜\left\{\Delta G_{k}\in C^{N_{T}\times N_{R}}:{∥\Delta G_{k}∥}^{2}\leq \upsilon_{k}^{2}\right\},\forall k,$$

where ${\bar{G}}_{k}$ is the channel estimate of the *k*-th energy vehicular receiver available at the base station while $\Delta G_{k}$ denotes the unknown channel uncertainty of the energy vehicular receiver *k*. For notational simplicity, a set $\Xi_{k}$ in Label (2), which includes all possible CSI uncertainties of energy vehicular receiver *k*. Moreover, the radius $\upsilon_{k}\geq 0$ denotes the size of the uncertainty region of the estimated CSI of the energy vehicular receiver *k*.

To provide vehicular secure communication and to facilitate EH at the desired information vehicular receiver, artificial noise signals and energy signals are generated at the base station. In particular, both signals could degrade the channels between the base station and the energy vehicular receivers and perform as an energy source for EH. As a result, we express the transmit signal vector x as

(3) $$x=ws_{I}+\sum_{k=1}^{K_{E}}v_{k}s_{E,k}+v_{0},$$

where $s_{I}\in C$ and $w\in C^{N_{T}\times 1}$ are the information-bearing signal and the corresponding beamforming vector for the information vehicular receiver, respectively. Without loss of generality, we suppose that $E\left\{|s_{I}{|}^{2}\right\}=1$. $v_{k}\in C^{N_{T}\times 1}$ and $s_{E}$ are the energy beamforming vector and the energy-bearing signal with $E\left\{|s_{E}{|}^{2}\right\}=1$, respectively. $v_{0}\in C^{N_{T}\times 1}$ represents the artificial noise vector generated by the base station to combat both passive and potential eavesdroppers, and we model it as $v_{0}∼\mathrm{CN}\left(0,V_{0}\right)$. With the transmit signal x, the received signal at information vehicular receiver and *k*-th energy vehicular receiver can be expressed as

(4) $$\begin{array}{cc} y_{I} & =h^{H}x+n_{I}\;and\;\\ y_{E,k} & =G_{k}^{H}x+n_{E,k},\forall k\in \left\{1,\cdots ,K_{E}\right\}, \end{array}$$

respectively, where $n_{I}∼\mathrm{CN}\left(0,\sigma_{I}^{2}\right)$ and $\sigma_{E,k}∼\mathrm{CN}\left(0,n_{E,k}^{2}I_{N_{R}}\right)$ represent the complex Gaussian noise at the information vehicular receiver and the *k*-th passive eavesdropper, respectively. $\sigma_{I}^{2}$ and $\sigma_{E,k}^{2}$ is the noise power of each other. In the considered networks, we exploit the energy and artificial noise signals transmitted by the base station to facilitate secure information transfer and charge both information vehicular receiver and energy vehicular receivers, respectively. In this paper, we adopt a practical nonlinear model. According to [35,36], the harvested energy at the *k*-th energy vehicular receiver can be modeled as:

(5) $$E_{E,k}^{\mathrm{Nonlinear}}=\frac{\Psi_{E,k}}{X_{E,k}}-Y_{E,k},$$

with

(6) $$\Psi_{E,k}=\frac{M_{E,k}}{1+\exp\left(-a_{E,k}\left(P_{E,k}\left(W,V,V_{0}\right)-b_{E,k}\right)\right)},$$

(7) $$\begin{array}{cc} P_{E,k}\left(W,V,V_{0}\right) & =∥G_{k}^{H}{w∥}^{2}+\sum_{k=1}^{K_{E}}∥G_{k}^{H}v_{k}{∥}^{2}+{∥G_{k}^{H}v_{0}∥}^{2}\\ & =Tr\left[G_{k}^{H}\left(W+V+V_{0}\right)G_{k}\right],\forall k, \end{array}$$

where $X_{E,k}=\frac{\exp\left(a_{E,k}b_{E,k}\right)}{1+\exp\left(a_{E,k}b_{E,k}\right)}$, $Y_{E,k}=\frac{M_{E,k}}{\exp\left(a_{E,k}b_{E,k}\right)}$, $W=ww^{H}$ and $V=\sum_{j=1}^{K_{E}}v_{j}v_{j}^{H}$. $P_{E,k}\left(W,V,V_{0}\right)$ denote the received RF power at the *k*-th energy vehicular receiver. Three parameters, namely, $M_{E,k}$, $a_{E,k}$ and $b_{E,k}$ in (6) are introduced to capture the joint effects of various nonlinear phenomena caused by hardware limitations in practical EH circuits. Furthermore, $M_{E,k}$ denotes the maximum power that can be collected by the EH circuit while $a_{E,k}$ and $b_{E,k}$ rely on serval hardware phenomena, such as the capacitance and diode turn-on voltage and the resistance.

As depicted in Figure 1, we adopt the power splitting architecture at the information vehicular receiver, the RF signals are divided into two streams by a power splitter in ρ, i.e., the 1−ρ part of the received signals is input into the EH receiver and the remaining ρ part is sent to the information receiver. Therefore, the received SINR can be expressed as

(8) $$\begin{array}{cc} \Gamma_{I} & =\frac{\rho |h^{H}{w|}^{2}}{\rho |h^{H}v_{0}{|}^{2}+\rho \sum_{i=1}^{K_{E}}{\left|h^{H}v_{i}\right|}^{2}+\sigma_{I}^{2}}\\ & =\frac{\rho Tr(HW)}{\rho Tr\left(HV_{0}\right)+\rho Tr\left(HV\right)+\sigma_{I}^{2}}. \end{array}$$

The input power at the information vehicular receiver can be written as

(9) $$\begin{array}{c} P_{I}\left(W,V,V_{0},\rho \right)=\left(1-\rho \right){\left|h^{H}w\right|}^{2}+\left(1-\rho \right)\left(|h^{H}v_{0}{|}^{2}+\sum_{i=1}^{K_{E}}{\left|h^{H}v_{i}\right|}^{2}\right)\\ =\left(1-\rho \right)Tr\left(\mathrm{HW}\right)+\left(1-\rho \right)\left[Tr\left(HV_{0}\right)+Tr\left(\mathrm{HV}\right)\right]. \end{array}$$

Here, we also adopt the nonlinear EH model, and then the harvested energy at the information vehicular receiver is given by

(10) $$\Phi_{I}\left(W,V,V_{0},\rho \right)=\frac{\Psi_{I}}{X_{I}}-Y_{I},$$

with

(11) $$\Psi_{I}=\frac{M_{I}}{1+\exp\left(-a_{I}\left(P_{I}\left(W,V,V_{0},\rho \right)-b_{I}\right)\right)},$$

where $X_{I}=\frac{\exp\left(a_{I}b_{I}\right)}{1+\exp\left(a_{I}b_{I}\right)}$ and $Y_{I}=\frac{M_{I}}{\exp\left(a_{I}b_{I}\right)}$. $M_{I}$ is a constant representing the maximum harvested power at the information vehicular receiver when the EH circuit is saturated. $a_{I}$ and $b_{I}$ are also constants associated with the detailed circuit specifications.

Without loss of generality, the energy vehicular receivers can also perform as eavesdroppers to decode the confidential information for the information vehicular receiver. Due to the fact that the energy signal $\sum_{k=1}^{K_{E}}v_{k}s_{E,k}$ is the only Gaussian pseudo-random sequence that is known at legitimate receivers (including both information vehicular receiver and energy vehicular receivers), we can thus perform interference cancellation at each energy vehicular receiver. As a result, the SINR of the *k*-th energy vehicular receiver is given by

(12) $$\Gamma_{E,k}=\frac{∥G_{k}^{H}{w∥}^{2}}{∥G_{k}^{2}v_{0}{∥}^{2}+\sigma_{E,k}^{2}}.$$

The optimal beamforming design policy, $\left\{W^{*},V^{*},V_{0}^{*},\rho^{*}\right\}$, for maximizing the minimum harvest energy among all the $K_{E}$ energy vehicular receivers, can be obtained by solving

(13) $$\begin{array}{cc} \max_{W,V,V_{0},\rho } & \min_{\Delta G_{k}\in \Xi_{k}}\;\frac{M_{E,k}}{X_{E,k}\left[1+\exp\left[-a_{E,k}\left(Tr\left({\left({\bar{G}}_{k}+\Delta G_{k}\right)}^{H}\left(W+V+V_{0}\right)\left({\bar{G}}_{k}+\Delta G_{k}\right)\right)-b_{E,k}\right)\right]\right]}-Y_{E,k}\\ s.t. & C1:\Gamma_{I}\geq r,\\ & C2:\max_{\Delta G_{k}\in \Xi_{k}}\frac{Tr\left({\left({\bar{G}}_{k}+\Delta G_{k}\right)}^{H}W\left({\bar{G}}_{k}+\Delta G_{k}\right)\right)}{\left({\left({\bar{G}}_{k}+\Delta G_{k}\right)}^{H}V_{0}\left({\bar{G}}_{k}+\Delta G_{k}\right)\right)}\leq r_{k},\forall k,\\ & C3:\Phi_{I}\left(W,V,V_{0},\rho \right)\geq E_{\min},\\ & C4:Tr\left(W+V+V_{0}\right)\leq P. \end{array}$$

The cost function in (13) takes into account the CSI uncertainty set $\Xi_{k}$ to provide robustness against CSI imperfection. In C1: *r* represents the minimum SINR of the information vehicular receiver for information decoding. Constraint C2 is imposed such that for a given CSI uncertainty set $\Xi_{k}$, the maximum received SINR at the passive eavesdropper *k* is not larger than the maximum tolerable received SINR $r_{k}$. In practice, $r\gg r_{k}>0$, $\forall k\in \left\{1,2,\cdots ,K_{E}\right\}$, to guarantee vehicular secure communication. Constraint C4 set the minimum required power transfer for the information vehicular receiver. Constraint C4 restricts the transmit power at the base station to account for the maximum power.

**Remark** **1.** *In our considered V2X-SWIPT network, the information vehicular receiver is assumed to have limited signal decoding capability and hardware complexity compared to the energy vehicular receivers. In order to ensure communication security, we adopt the worse-case assumption that the information vehicular receiver is equipped with a single antenna while all energy vehicular receivers is equipped with multiple antennas.*

## 3. Solution of the Optimization Problem

It can be observed that the optimization problem (13) is a non-convex quadratically constrained programming that involves semi-infinite programming. Particularly, the non-convexity with respect to the information beamforming matrix W and the power splitting ratio ρ is owing to constraints (C1) and (C3). In addition, both the objective function in (13) and constraint (C2) involve infinitely many possibilities due to the CSI uncertainties. In general, there are no standard methods for solving the non-convex optimization problem. In extreme cases, we require an exhaustive search method to get the optimal solution that is computationally intractable even for a moderate network size. To deduce an efficient beamforming design algorithm for the considered optimization problem, we first recast the optimization problem (13) as a semi-definite programming (SDP) problem to avoid the non-convexity associated with the objective function, and constraints (C1) and (C3). Next, the infinite number of constraints is converted into an equivalent finite number of constraints. The performance of the reformulated optimization problem is the lower bound for the original optimization problem. Finally, we apply semi-definite programming relaxation (SDR) to obtain the globally optimal beamforming for the reformulated optimization problem. In practice, the considered optimization problem may be infeasible when the channels are in unfavourable conditions. However, in the following, we suppose that the problem is always feasible for investigating the design of different beamforming schemes.

### 3.1. Semi-Definite Programming Relaxation

In order to solve the optimization problem (13), we introduce a slack variable t≥0. With the slack variable *t*, the problem (13) can be equivalently formulated as the following optimization problem:

(14) $$\begin{array}{cc} \max_{W,V,V_{0},\rho ,t} & t\\ \;s.t. & C1:Tr\left[\left(W-rV_{0}-rV\right)H\right]\geq \frac{\sigma_{I}^{2}r}{\rho },\\ & C2:\max_{\Delta G_{k}\in \Xi_{k}}Tr\left[G_{k}^{H}\left(W-r_{k}V_{0}\right)G_{k}\right]\leq r_{k}\sigma_{E,k}^{2},\forall k,\\ & C3:\Phi_{I}\left(W,V,V_{0},\rho \right)\geq E_{\min},\\ & C4:Tr\left(W+V+V_{0}\right)\leq P,\\ & C5:\min_{\Delta G_{k}\in \Xi_{k}}\;E_{E,k}^{\mathrm{Nonlinear}}\geq t,\forall k,\\ & C6:0\leq \rho \leq 1,\;\;\;\;\;\;C7:W,V,V_{0}⪰0,\\ & C8:\mathrm{Rank}\left(W\right)=1. \end{array}$$

To facilitate the optimization problem (14), another slack variable $\tau_{k}$, $\forall k\in \left\{1,\cdots ,K_{E}\right\}$ is also introduced, we can rewrite the problem (14) as

(15) $$\begin{array}{cc} \max_{W,V,V_{0},\rho ,t,\tau } & t\\ s.t. & C1:Tr\left[\left(W-rV_{0}-rV\right)H\right]\geq \frac{\sigma_{I}^{2}r}{\rho },\\ & C2:\max_{\Delta G_{k}\in \Xi_{k}}Tr\left[G_{k}^{H}\left(W-r_{k}V_{0}\right)G_{k}\right]\leq r_{k}\sigma_{E,k}^{2},\forall k,\\ & C3:Tr\left[\left(W+V+V_{0}\right)H\right]\geq \frac{\omega_{I}}{1-\rho },\\ & C4:Tr\left(W+V+V_{0}\right)\leq P,\\ & C5:\frac{M_{E,k}}{1+\exp\left(-a_{E,k}\left(\tau_{E,k}-b_{E,k}\right)\right)}\geq X_{E,k}\left(Y_{E,k}+t\right),\forall k,\\ & C6:0\leq \rho \leq 1,\;\;\;\;\;\;C7:W,V,V_{0}⪰0,\\ & C8:\mathrm{Rank}\left(W\right)=1,\\ & C9:\min_{\Delta G_{k}\in \Xi_{k}}Tr\left(G_{k}^{H}\left(W+V+V_{0}\right)G_{k}\right)\geq \tau_{E,k}, \end{array}$$

where $\omega_{I}=b_{I}-\frac{\ln\left\{\frac{M_{I}}{\left(E_{\min}+Y_{I}\right)X_{I}}-1\right\}}{a_{I}}$; W⪰0, $W\in H_{+}^{N_{T}}$ and rank(W)=1 are imposed to guarantee that $W=ww^{H}$ holds after optimizing W. After some manipulations, it can be easily known that constraints (C1) and (C3) are jointly convex with regard to $\left\{W,V,V_{0},\rho \right\}$. Then, we deal with constraints (C2), (C5) and (C9). Although constraints (C1) and (C3) are jointly convex with regard to the optimization variables, they are semi-infinite constraints that are generally intractable for beamforming scheme design. To facilitate this, we can transform them into linear matrix inequalities using the following lemma.

**Lemma** **1.** *(S-Procedure) Let $f_{m}\left(x\right)=x^{H}A_{m}x+2Re\left\{b_{m}^{H}x\right\}+c_{m}$, $m\in \left\{1,2\right\}$, where $x\in C^{N\times 1}$, $A_{m}\in H^{N\times N}$, $b_{m}\in C^{N\times 1}$ and $c_{m}\in R$. Then, the expression $f_{1}\left(x\right)\leq 0\Rightarrow f_{2}\left(x\right)$ holds if and only if there exists a λ≥0 such that we get:*

(16) $$\lambda \left[\begin{array}{cc} A_{1} & b_{1}\\ b_{1}^{H} & c_{1} \end{array}\right]-\left[\begin{array}{cc} A_{2} & b_{2}\\ b_{2}^{H} & c_{2} \end{array}\right]⪰0$$

*provided that there exsists a vector $\hat{x}$ such that we obtain $f_{i}\left(\hat{x}\right)<0$.*

As a result, we can perform Lemma 1 to constraint (C2). Particularly, we substitute $G_{k}={\bar{G}}_{k}+\Delta G_{k}$ in constraint C2. Hence, the implication

(17) $$\begin{array}{c} \Delta g_{k}^{H}\Delta g_{k}\leq v_{k}^{2}\Rightarrow ,\\ \Delta g_{k}^{H}\Delta_{2}\Delta g_{k}+2Re\left\{{\bar{g}}_{k}^{H}\Delta_{2}\Delta g_{k}\right\}+{\bar{g}}_{k}^{H}\Delta_{2}{\bar{g}}_{k}-r_{k}\sigma_{E,k}^{2}\leq 0 \end{array}$$

holds if and only if there exist ${\bar{\lambda }}_{k}\geq 0$, $k\in \left\{1,2,\cdots ,K_{E}\right\}$, such that the following linear matrix inequalities hold:

(18) $$\begin{array}{cc} C2: & N_{E,k}\left(W,V_{0},{\bar{\lambda }}_{k}\right)\\ & =\left[\begin{array}{cc} {\bar{\lambda }}_{k}I_{N_{T}N_{R}}-\Delta_{2} & -\Delta_{2}{\bar{g}}_{k}\\ {\bar{g}}_{k}^{H}\Delta_{2} & -{\bar{g}}_{k}^{H}\Delta_{2}{\bar{g}}_{k}-v_{k}^{2}{\bar{\lambda }}_{k}+r_{k}\sigma_{E,k}^{2} \end{array}\right]⪰0, \end{array}$$

where $\bar{W}=I_{N_{R}}⊗W$, ${\bar{V}}_{0}=I_{N_{R}}⊗V_{0}$, $\Delta g_{k}=vec\left(\Delta G_{k}\right)$, ${\bar{g}}_{k}=vec\left({\bar{G}}_{k}\right)$ and $\Delta_{2}=\bar{W}-r_{k}{\bar{V}}_{0}$. Similarly, by applying Lemma 1, we can equivalently write the constraint (C9) as

(19) $$\begin{array}{cc} C9: & T_{E,k}\left(W,V_{0},V,{\bar{\mu }}_{k},t\right)\\ & =\left[\begin{array}{cc} {\bar{\mu }}_{k}I_{N_{T}N_{R}}+\Delta_{3} & \Delta_{3}{\bar{g}}_{k}\\ {\bar{g}}_{k}^{H}\Delta_{3} & {\bar{g}}_{k}^{H}\Delta_{3}{\bar{g}}_{k}-v_{k}^{2}{\bar{\mu }}_{k}-\tau_{E,k} \end{array}\right]⪰0 \end{array}$$

for ${\bar{\mu }}_{k}\geq 0$, $k\in \left\{1,2,\cdots ,K_{E}\right\}$, where $\bar{V}=I_{N_{R}}⊗V$ and $\Delta_{3}=\bar{W}+\bar{V}+{\bar{V}}_{0}$. Therefore, substituting (18) and (19) back into (15)

(20) $$\begin{array}{cc} & \max_{W,V,V_{0},\rho ,t,\tau_{E,k},\bar{\lambda },\bar{\mu }}\;t\\ s.t. & C1,\;C3,\;C4,\;C5,\;C6,C7,\\ & C2:N_{E,k}\left(W,V_{0},{\bar{\lambda }}_{k}\right)⪰0,\forall k,\\ & C8:\;\mathrm{rank}(W)=1,\\ & C9:T_{E,k}\left(W,V_{0},V,{\bar{\mu }}_{k},t\right)⪰0,\forall k,\\ & C10:\;{\bar{\lambda }}_{k}\geq 0,\;{\bar{\mu }}_{k}\geq 0,\;\forall k, \end{array}$$

where $\bar{\lambda }$ and $\bar{\mu }$ denote auxiliary optimization variable vectors, whose elements ${\bar{\lambda }}_{k}$, $k\in \left\{1,2,\cdots ,K_{E}\right\}$, and ${\bar{\mu }}_{k}$, $k\in \left\{1,2,\cdots ,K_{E}\right\}$ were introduced in (18) and (19), respectively. Now, (C8): rank(W)=1 is the main obstacle in solving the optimization problem (20). By relaxing constraint (C8), i.e., dropping the rank constraint from the optimization problem (20), we can reexpress the optimization problem (20) as follows:

(21) $$\begin{array}{c} \max_{W,V,V_{0},\rho ,t,\tau_{E,k},\bar{\lambda },\bar{\mu }}\;t\\ s.t.\;\;C1,C2,C3,C4,C5,C6,C7,C9,C10. \end{array}$$

It is worth noting that the relaxed optimization problem (21) becomes a convex SDP problem that can be solved efficiently by a numerical solver such as SDPT3 and SeDuMi. Based on the basic principles of convex optimization theory, if the obtained solution W for optimization problem (21) admits a rank-one matrix, then W is the optimal solution of the optimization problem (20). Then, we can obtain the optimal information vector w by performing eigenvalue decomposition on W. However, in some cases, the rank of the obtained W is higher than one, thus the resulting solution may not the optimal solution for the relaxed problem. In the following, a theorem that reveals the tightness of the relaxed optimization problem (21) is first introduced. Then, a method for constructing an optimal solution for the relaxed optimization problem (21) with rank(W)=1 is proposed.

### 3.2. Optimality Conditions for SDP Relaxation

In this subsection, the tightness of the proposed SDP relaxation in (20) is revealed. Before introducing the theorem, a crucial expression, which is a cornerstone in revealing the tightness of our proposed SDP relaxation optimization problem, is defined as

(22) $$T^{*}=-\alpha^{*}I_{N_{T}}+\sum_{k=1}^{K_{E}}\sum_{n=1}^{N_{R}}\left(J_{E,k}^{\left(n,n)(*\right)}-K_{E,k}^{\left(n,n)(*\right)}\right)$$

and $\tilde{r}=\mathrm{rank}\left(T^{*}\right)$, where $J_{E,k}^{\left(n,n)(*\right)}\in H_{+}^{N_{T}}$ and $K_{E,k}^{\left(n,n)(*\right)}\in H_{+}^{N_{T}}$ denote the *n*-th entry matrices on the diagonal of ${\tilde{G}}_{k}^{H}X_{k}{\tilde{G}}_{k}\in H_{+}^{N_{T}N_{R}}$ and ${\tilde{G}}_{k}^{H}Y_{k}{\tilde{G}}_{k}\in H_{+}^{N_{T}N_{R}}$, respectively; where ${\tilde{G}}_{k}=\left[I_{N_{T}N_{R}}\;{\bar{g}}_{k}\right]$, and $X_{k}$ and $Y_{k}$ represent the Lagrange dual variables corresponding to $N_{E,k}\left(W,V_{0},{\bar{\lambda }}_{k}\right)$ and $T_{E,k}\left(W,V_{0},V,{\bar{\mu }}_{k},t\right)$, respectively. Moreover, $N_{1}\in C^{N_{T}\times \left(N_{T}-\tilde{r}\right)}$ is modeled as the orthogonal basis of the null space of $T^{*}$ and $\pi_{1,n}\in c^{n_{t}\times 1}$, $1\leq n\leq N_{T}-\tilde{r}$ is the *n*-th column of the matrix $N_{1}$. Based on this, we give the following theorem.

**Theorem** **1.** *The optimal solution $\left\{W^{*},V^{*},V_{0}^{*},\rho^{*},t^{*}\right\}$ of the optimization problem (20) is characterized as the following conditions:*

*1. The optimal solution $W^{*}$ can be formulated as*

(23) $$W^{*}=\sum_{n=1}^{N_{T}-\tilde{r}}b_{n}\pi_{1,n}\pi_{1,n}^{H}+a\iota \iota^{H},$$

*where $b_{n}\geq 0$, ∀n and $\iota \in C^{N_{T}\times 1}$, ${∥\iota ∥}_{2}=1$ satisfies $\iota^{H}N_{1}=0$.*

*2. If the solution $W^{*}$ given in (23) has $\mathrm{rank}(W^{*})>1$, i.e., there is at least an n such that $b_{n}>0$, then we have the following alternative solution*

(24) $${\tilde{W}}^{*}=W^{*}-\sum_{n=1}^{N_{T}-\tilde{r}}b_{n}\pi_{1,n}\pi_{1,n}^{H}=a\iota \iota^{H},$$

(25) $${\tilde{V}}^{*}=V^{*}+\sum_{n=1}^{N_{T}-\tilde{r}}b_{n}\pi_{1,n}\pi_{1,n}^{H},$$

(26) $${\tilde{V}}_{0}=V_{0}^{*},\;\;{\tilde{\rho }}^{*}=\rho^{*},\;\;{\tilde{t}}^{*}=t^{*},$$

*with $rank\left({\tilde{W}}^{*}\right)=1$ serving as the optimal solution of optimization problem (20).*

**Proof.**  The Lagrangian dual function of problem (20) can be modeled as

(27) $$\begin{array}{c} L(W,V,V_{0},X_{k},Y_{k},\alpha ,\beta ,\gamma ,\zeta_{k},\Theta ,\Xi ,\Omega ,\rho ,t,\tau_{E,k},{\bar{\lambda }}_{k},{\bar{\mu }}_{k})\\ =t+\sum_{k=1}^{K_{E}}Tr\left(X_{k}N_{E,k}\left(W,V_{0},{\bar{\lambda }}_{k}\right)\right)\\ +\alpha \left[P-Tr\left(W+V+V_{0}\right)\right]\\ +\beta [Tr\left(\left(W-rV-rV_{0}\right)H\right)-\frac{\sigma_{I}^{2}r}{\rho }]\\ +\gamma \left[Tr\left(\left(W+V+V_{0}\right)H\right)-\frac{\omega_{I}}{1-\rho }\right]\\ +\sum_{k=1}^{K_{E}}Tr\left(Y_{k}T_{E,k}\left(W,V_{0},V,{\bar{\mu }}_{k},t\right)\right)\\ +\sum_{k=1}^{K_{E}}\zeta_{k}\left[\frac{M_{E,k}}{1+\exp\left(-a_{E,k}\left(\tau_{E,k}-b_{E,k}\right)\right)}-X_{E,k}\left(Y_{E,k}+t\right)\right]\\ +Tr\left(\Theta W\right)+Tr\left(\Xi V\right)+Tr\left(\Omega V_{0}\right), \end{array}$$

where $X_{k}⪰0$, ∀k, $Y_{k}⪰0$, ∀k, α≥0, β≥0, γ≥0, $\zeta_{k}\geq 0$, ∀k are the dual variables for constraints C2, C9, C4, C1, C3, and C5, respectively. $\Theta \in H_{+}^{N}$, $\Xi \in H_{+}^{N}$ and $\Omega \in H_{+}^{N}$ are the dual variables with respect to parameters W, V and $V_{0}$, respectively. Let ${\tilde{G}}_{k}=\left[I_{N_{T}N_{R}}\;\;{\bar{g}}_{k}\right]$, $\Lambda_{E,k}=\left[\begin{array}{cc} {\bar{\mu }}_{k}I_{N_{T}N_{R}} & 0\\ 0 & -v_{k}^{2}{\bar{\mu }}_{k}-\tau_{E,k} \end{array}\right]$, ${\bar{\Lambda }}_{E,k}=\left[\begin{array}{cc} {\bar{\lambda }}_{k}I_{N_{T}N_{R}} & 0\\ 0 & -v_{k}^{2}{\bar{\lambda }}_{k}+r_{k}\sigma_{E}^{2} \end{array}\right]$, we can rewrite the $N_{E,k}\left(W,V_{0},{\bar{\lambda }}_{k}\right)$ and $T_{E,k}\left(W,V_{0},V,{\bar{\mu }}_{k},t\right)$ as follows:

(28) $$\begin{array}{c} N_{E,k}\left(W,V_{0},{\bar{\lambda }}_{k}\right)={\tilde{G}}_{k}^{H}\left(r_{k}{\bar{V}}_{0}-\bar{W}\right){\tilde{G}}_{k}+{\bar{\Lambda }}_{E,k},\\ T_{E,k}\left(W,V_{0},V,{\bar{\mu }}_{k},t\right)={\tilde{G}}_{k}^{H}\left(\bar{W}+\bar{V}+{\bar{V}}_{0}\right){\tilde{G}}_{k}+\Lambda_{E,k}. \end{array}$$

For the convenience of analyzing the construction of the resulting solution, we must write back the variable $\bar{W}$, $\bar{V}$, and ${\bar{V}}_{0}$ into their primal styles. Hence, we have

(29) $$\begin{array}{cc} {\tilde{G}}_{k}^{H}X_{k}{\tilde{G}}_{k} & =\left[\begin{array}{cccc} K_{E,k}^{\left(1,1\right)} & K_{E,k}^{\left(1,2\right)} & \cdots & K_{E,k}^{\left(1,N_{R}\right)}\\ K_{E,k}^{\left(2,1\right)} & K_{E,k}^{\left(2,2\right)} & \cdots & K_{E,k}^{\left(1,N_{R}\right)}\\ \vdots & \vdots & \ddots & \vdots \\ K_{E,k}^{\left(N_{R},1\right)} & K_{E,k}^{\left(N_{R},2\right)} & \cdots & K_{E,k}^{\left(N_{R},N_{R}\right)} \end{array}\right]\in H_{+}^{N_{T}N_{R}},\\ {\tilde{G}}_{k}^{H}Y_{k}{\tilde{G}}_{k} & =\left[\begin{array}{cccc} J_{E,k}^{\left(1,1\right)} & J_{E,k}^{\left(1,2\right)} & \cdots & J_{E,k}^{\left(1,N_{R}\right)}\\ J_{E,k}^{\left(2,1\right)} & J_{E,k}^{\left(2,2\right)} & \cdots & J_{E,k}^{\left(1,N_{R}\right)}\\ \vdots & \vdots & \ddots & \vdots \\ J_{E,k}^{\left(N_{R},1\right)} & J_{E,k}^{\left(N_{R},2\right)} & \cdots & J_{E,k}^{\left(N_{R},N_{R}\right)} \end{array}\right]\in H_{+}^{N_{T}N_{R}},\\ & J_{E,k}^{\left(k,k\right)}\in H_{+}^{N_{R}},K_{E,k}^{\left(k,k\right)}\in H_{+}^{N_{R}}. \end{array}$$

Then, by utilizing (27)–(29), the Lagrange dual function can be rewritten as

(30) $$\begin{array}{c} L(W,V,V_{0},X_{k},Y_{k},\alpha ,\beta ,\gamma ,\zeta_{k},\Theta ,\Xi ,\Omega ,\rho ,t,\tau_{E,k},{\bar{\lambda }}_{k},{\bar{\mu }}_{k})\\ =t+\sum_{k=1}^{K_{E}}Tr\left[X_{k}{\bar{\Lambda }}_{E,k}+\sum_{n=1}^{N_{R}}\left(r_{k}V_{0}-W\right)K_{E,k}^{\left(n,n\right)}\right]\\ +\sum_{k=1}^{K_{E}}Tr\left[Y_{k}\Lambda_{E,k}+\sum_{n=1}^{N_{R}}\left(W+V+V_{0}\right)J_{E,k}^{\left(n,n\right)}\right]\\ +\alpha \left[P-Tr\left(W+V+V_{0}\right)\right]+\beta [Tr\left(\left(W-rV-rV_{0}\right)H\right)-\frac{\sigma_{I}^{2}r}{\rho }]\\ +\gamma \left[Tr\left(\left(W+V+V_{0}\right)H\right)-\frac{\omega_{I}}{1-\rho }\right]+Tr\left(\Theta W\right)+Tr\left(\Xi V\right)+Tr\left(\Omega V_{0}\right)\\ +\sum_{k=1}^{K_{E}}\zeta_{k}\left[\frac{M_{E,k}}{1+\exp\left(-a_{E,k}\left(\tau_{E,k}-b_{E,k}\right)\right)}-X_{E,k}\left(Y_{E,k}+t\right)\right]. \end{array}$$

It can be verified that the relax optimization problem (20) is jointly convex with regard to the primal variables and satisfies the Slater’s constraint qualification. Therefore, there is no gap between the primal optimization problem and dual optimization problem. That is to say, strong duality holds and solving the dual optimization problem is equivalent to solving the primal problem. Considering (30), the dual optimization problem is expressed as:

(31) $$\min_{\left\{X_{k}⪰0\right\},\left\{Y_{k}⪰0\right\},\Theta ,\Xi ,\Omega ⪰0,\alpha ,\beta ,\gamma \geq 0,\left\{\zeta_{k}\geq 0\right\}}\max_{W,V,V_{0},\rho }L\left(\Pi \right),$$

where $\Pi ≜\left\{W,V,V_{0},X_{k},Y_{k},\alpha ,\beta ,\gamma ,\zeta_{k},\Theta ,\Xi ,\Omega ,\rho ,t,\tau_{E,k},{\bar{\lambda }}_{k},{\bar{\mu }}_{k}\right\}$. From (31), we have $\left\{X_{k}^{*},Y_{k}^{*},\Theta^{*},\Xi^{*},\Omega^{*},\alpha^{*},\beta^{*},\gamma^{*},\zeta_{k}^{*}\right\}$ and $\left\{W^{*},V^{*},V_{0}^{*},\rho^{*},\tau_{E,k}^{*},t^{*}\right\}$ as the dual and the primal optimal solution of (20) and (31), respectively. Considering (31), the Karush–Kuhn–Tucker (KKT) conditions for the optimal $W^{*}$ are given by

(32) $$\Theta^{*}⪰0,\alpha \geq 0,\beta \geq 0,\gamma \geq 0,$$

(33) $$\Theta^{*}W^{*}=0,$$

(34) $$\left[\sum_{k=1}^{K_{E}}\sum_{n=1}^{N_{R}}\left(J_{E,k}^{\left(n,n)(*\right)}-K_{E,k}^{\left(n,n)(*\right)}\right)+\left(\beta^{*}+\gamma^{*}\right)H-\alpha^{*}I_{N_{T}}\right]W^{*}+\Theta^{*}=0,$$

(35) $$\frac{\partial L(\Pi )}{\partial \rho^{*}}=0\Rightarrow \;\rho^{*}=\frac{\sqrt{\beta^{*}r\sigma_{I}^{2}}}{\sqrt{\gamma^{*}\omega_{I}}+\sqrt{\beta^{*}r\sigma_{I}^{2}}}.$$

For notational simplicity, we define

(36) $$\begin{array}{cc} R^{*} & =\sum_{k=1}^{K_{E}}\sum_{n=1}^{N_{R}}\left(J_{E,k}^{\left(n,n)(*\right)}-K_{E,k}^{\left(n,n)(*\right)}\right)+\left(\beta^{*}+\gamma^{*}\right)H-\alpha^{*}I_{N_{T}}. \end{array}$$

Then, we have

(37) $$R^{*}=T^{*}+\left(\beta^{*}+\gamma^{*}\right)H.$$

However, in general, we define $\tilde{r}=\mathrm{rank}\left(T^{*}\right)$. Then, we consider two cases of $\tilde{r}$ with the aim of analyzing $R^{*}$. Firstly, it is assumed that $\tilde{r}=N_{T}$, i.e., the matrix $T^{*}$ is full rank. Upon that, we obtain

(38) $$\begin{array}{c} \mathrm{rank}\left(R^{*}\right)=\mathrm{rank}\left(T^{*}+\left(\beta^{*}+\gamma^{*}\right)H\right),\\ \geq \mathrm{rank}\left(T^{*}\right)-\mathrm{rank}\left(\left(\beta^{*}+\gamma^{*}\right)H\right)=N_{T}-1. \end{array}$$

However, if $\mathrm{rank}\left(R^{*}\right)=N_{T}$, then according to (33) and (34) it follows that $W^{*}=0$, which, of course, cannot be the optimal solution to optimization problem (20). Thus, we obtain $\mathrm{rank}\left(T^{*}\right)=N_{T}-1$ and get the optimal solution $W^{*}=a\iota \iota^{H}$, a≥0 if $\tilde{r}=N_{T}$ where ι lies in the null space of $R^{*}$ with unit norm. Next, we consider the cast where $T^{*}$ is rank defective, i.e., $\tilde{r}\leq N_{T}$. In this case, the matrix $N_{1}$ is modeled as the standard orthogonal basis that spans the null space of $T^{*}$, i.e., $T^{*}N_{1}=0$. Let $\pi_{1,n}$ represent the *n*-th column of $N_{1}$, $1\leq n\leq N_{T}-\tilde{r}$. The following equation holds:

(39) $$\begin{array}{c} \pi_{1,n}^{H}R^{*}\pi_{1,n}=\pi_{1,n}^{H}\left(T^{*}+\left(\beta^{*}+\gamma^{*}\right)H\right)\pi_{1,n}\\ =\left(\beta^{*}+\gamma^{*}\right)\pi_{1,n}^{H}H\pi_{1,n}=\left(\beta^{*}+\gamma^{*}\right)\pi_{1,n}^{H}hh^{H}\pi_{1,n}. \end{array}$$

Since $R^{*}⪯0$ and $|\pi_{1,n}^{H}h|\geq 0$, it follows that

(40) $$R^{*}N_{1}=0\;and\;HN_{1}=0.$$

Furthermore, according to (37), another inequality is achieved, i.e., $\mathrm{rank}\left(R^{*}\right)\geq \mathrm{rank}\left(T^{*}\right)-\mathrm{rank}\left(H\right)=\tilde{r}-1$. We define $\Omega_{1}$ as the orthogonal basis for the null space of $R^{*}$; it then yields

(41) $$\mathrm{rank}\left(\Omega_{1}\right)=N_{T}-\mathrm{rank}\left(R^{*}\right)\leq N_{T}-\tilde{r}+1.$$

Next, we come to show that $\mathrm{rank}\left(\Omega_{1}\right)=N_{T}-\tilde{r}+1$. With (40), $N_{1}$ spans $N_{T}-\tilde{r}$ orthogonal dimensions of the null space of $A^{*}$, i.e., $\mathrm{rank}\left(\Omega_{1}\right)\geq N_{T}-\tilde{r}$. Assume that $\mathrm{rank}\left(\Omega_{1}\right)=N_{T}-\tilde{r}$; then, we get $\Omega_{1}=N_{1}$ and express $W^{*}$ as $W^{*}=\sum_{n=1}^{N_{T}-\tilde{r}}b_{n}\pi_{1,n}\pi_{1,n}^{H}$, where $b_{n}\geq 0$, ∀n. Moreover, in this case, there is no information transferred to information receiver according to (40). Therefore, according to (41), there exists only one single subspace spanned by unit norm vector ι that also satisfies $N_{1}\iota =0$. Therefore, we have

(42) $$\Omega_{1}=\left[N_{1}\;\;\iota \right]$$

and $\mathrm{rank}\left(\Omega_{1}\right)=N_{T}-1+\tilde{r}$. Furthermore, according to (33) and (34), any optimal solution $W^{*}$ for optimization problem (20) can be modeled as

(43) $$W^{*}=\sum_{n=1}^{N_{T}-\tilde{r}}b_{n}\pi_{1,n}\pi_{1,n}^{H}+a\iota \iota^{H},$$

where $b_{n}\geq 0$, ∀n, and a≥0. The first part of Theorem 1 is thus proved.

Next, we prove the second part of Theorem 1. Suppose we obtained the optimal solution $\left\{W^{*},V^{*},V_{0}^{*},\rho^{*},t^{*}\right\}$, where $W^{*}$ is given by (23) and $\mathrm{rank}(W^{*})>1$. Then, the new alternative solution $\left\{{\tilde{W}}^{*},{\tilde{V}}^{*},{\tilde{V}}_{0}^{*},{\tilde{\rho }}^{*},{\tilde{t}}^{*}\right\}$ can be given in (23)–(26) and has the following properties:

(44) $$\begin{array}{c} Tr\left[\left({\tilde{W}}^{*}-r{\tilde{V}}^{*}-r{\tilde{V}}_{0}^{*}\right)H\right]\\ =Tr\left[\left(W^{*}-rV^{*}-rV_{0}^{*}-\left(r+1\right)\sum_{n=1}^{N_{T}-\tilde{r}}b_{n}\pi_{1,n}\pi_{1,n}^{H}\right)H\right]\\ =Tr\left[\left(W^{*}-V^{*}-V_{0}^{*}\right)H\right]\geq \frac{\sigma_{I}^{2}r}{{\tilde{\rho }}^{*}}, \end{array}$$

(45) $$\begin{array}{c} Tr\left[G_{k}^{H}\left({\tilde{W}}^{*}-r_{k}{\tilde{V}}_{0}^{*}\right)G_{k}\right]\\ =Tr\left[G_{k}^{H}\left(W^{*}-\sum_{n=1}^{N_{T}-\tilde{r}}b_{n}\pi_{1,n}\pi_{1,n}^{H}-r_{k}V^{*}\right)G_{k}\right]\\ \leq Tr\left[G_{k}^{H}\left(W^{*}-r_{k}V^{*}\right)G_{k}\right]\leq r_{k}\sigma_{E,k}^{2}, \end{array}$$

(46) $$\begin{array}{c} Tr\left[\left({\tilde{W}}^{*}+{\tilde{V}}^{*}+{\tilde{V}}_{0}^{*}\right)H\right]\\ =Tr\left[\left(W^{*}+V^{*}+V_{0}^{*}\right)H\right]\geq \frac{\omega_{I}}{1-{\tilde{\rho }}^{*}}, \end{array}$$

(47) $$\begin{array}{c} Tr\left[G_{k}^{H}\left({\tilde{W}}^{*}+{\tilde{V}}^{*}+{\tilde{V}}_{0}^{*}\right)G_{k}\right]\\ =Tr\left[G_{k}^{H}\left(W^{*}+V^{*}+V_{0}^{*}\right)G_{k}\right]\geq {\tilde{t}}^{*},\;\forall k, \end{array}$$

(48) $$Tr\left({\tilde{W}}^{*}+{\tilde{V}}^{*}+{\tilde{V}}_{0}^{*}\right)=Tr\left(W^{*}+V^{*}+V_{0}^{*}\right)\leq P,$$

(49) $${\tilde{W}}^{*}⪰0,\;\;{\tilde{V}}^{*}⪰0,\;\;{\tilde{V}}_{0}^{*}⪰0.$$

The properties from (44) to (49) indicate that the new solution $\left\{{\tilde{W}}^{*},{\tilde{V}}^{*},{\tilde{V}}_{0}^{*},{\tilde{\rho }}^{*},{\tilde{t}}^{*}\right\}$ can achieve the same optimal value as $\left\{W^{*},V^{*},V_{0}^{*},\rho^{*},t^{*}\right\}$ while (44)–(49) demonstrate that the new optimal solution satisfies all the constraints of primal problem (20) with $\mathrm{rank}({\tilde{W}}^{*})=1$. Theorem 1 is thus proved. ☐

With Theorem 1, we can achieve the global optimal solution of the optimization problem (13) with rank(W)=1 as follows. First, we solve the SDP relaxation optimization problem (20) via CVX and obtain the solution $\left\{W^{*},V^{*},V_{0}^{*},\rho^{*},t^{*}\right\}$. If the rank of the obtained $W^{*}$ equals one, then $\left\{W^{*},V^{*},V_{0}^{*},\rho^{*},t^{*}\right\}$ will be the optimal solution to problem (20). Otherwise, if the rank of $W^{*}$ has a larger rank than one, a new optimal solution $\left\{{\tilde{W}}^{*},{\tilde{V}}^{*},{\tilde{V}}_{0}^{*},{\tilde{\rho }}^{*},{\tilde{t}}^{*}\right\}$ with $\mathrm{rank}({\tilde{W}}^{*})=1$ can be constructed according to (23)–(26). Then, $\left\{{\tilde{W}}^{*},{\tilde{V}}^{*},{\tilde{V}}_{0}^{*},{\tilde{\rho }}^{*},{\tilde{t}}^{*}\right\}$ will be the optimal solution to (20). Hence, dropping the rank-one constraint in (15) results in no loss of optimality to (13).

## 4. Simulation Results

In this section, we provide simulation results to validate the performance of our proposed robust beamforming design algorithm. The considered vehicular secure downlink channel in Section 2 for Rayleigh flat-fading environments with zero-mean and unit variance is considered. Unless specified otherwise, it is assumed that there are $K_{E}=3$ energy vehicular receivers. Indeed, we have some other configurations that can be employed with a different number of energy vehicular receivers, transmit antennas and receiver antennas; however, the simulation results will be similar, the only difference is the computation complexity. In our simulations, we set $N_{T}=6$, $N_{R}=3$, *r* = 10 dB, $r_{k}=\bar{r}$ = 0 dB, P= 25 dBm, $E_{\min}\;=0$ mW, $\sigma_{I}^{2}=\sigma_{E,k}^{2}=\sigma^{2}=-20$ dBm. For the nonlinear EH model, we set: $M_{I}=M_{E,k}=M=24$ mW, $a_{I}=a_{E,k}=\bar{a}=1500$, $b_{I}=b_{E,k}=\bar{b}=0.014$. To the imperfect CSI between the base station and energy vehicular receivers, $d_{E,k}^{2}=\frac{\upsilon_{k}^{2}}{∥{\bar{G}}_{k}{∥}^{2}}$ is defined as the channel uncertainty ratio to demonstrate the *k*th energy vehicular receiver’s channel estimate error. Without loss of generality, it is assumed that $d_{k}=\delta_{E}$. We solve the optimization problem (21) and compute the average minimum harvested energy power per energy vehicular receiver by averaging over 1000 channel realizations.

**Figure 2:**
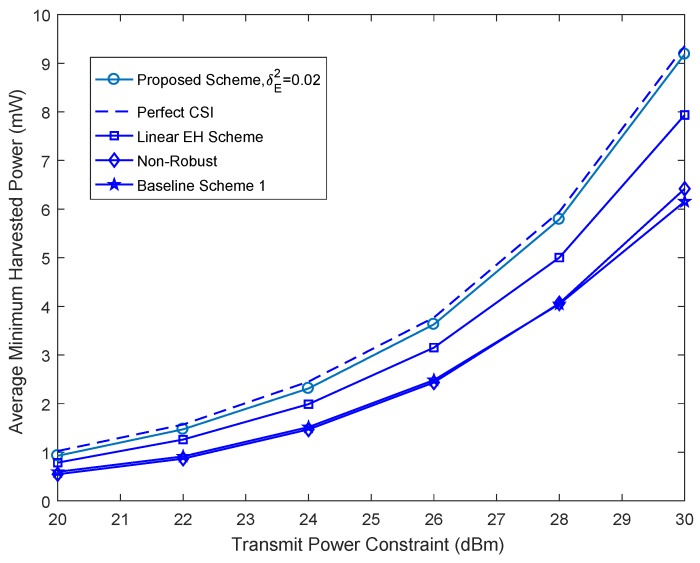
Average minimum harvested power per energy vehicular receiver versus total transmit power, *P*.

Figure 2 depicts the average minimum harvested energy power per energy vehicular receiver versus the maximum power of the base station, *P*, for $K_{E}=3$ energy vehicular receivers, $\delta_{E}^{2}=0.02$, and different beamforming design schemes. We learn that the average minimum harvested energy power per energy vehicular receiver of the proposed beamforming design scheme is a monotonically increasing function of *P*. This is attributed to the fact that a higher harvested energy power is necessary for satisfying constraint C4 when the maximum transmit power requirement of *P* becomes more stringent. For a comprehensive comparison, Figure 2 also contains the average minimum harvested energy power per energy vehicular receiver of the proposed algorithm that the base station has perfect CSI, the non-robust scheme, the linear EH scheme and two baseline beamforming design schemes. For the non-robust scheme, we treat the estimated channel ${\bar{G}}_{k}$, $\forall k\in \left\{1,2,\cdots ,K_{E}\right\}$ as true CSI, and then optimize $\left\{W,V,V_{0},\rho \right\}$ jointly in (21) according to ${\bar{G}}_{k}$, $\forall k\in \left\{1,2,\cdots ,K_{E}\right\}$. For the linear EH scheme, we adopt the existing linear EH model for the beamforming design algorithm. In particular, $\left\{W,V,V_{0},\rho \right\}$ optimizes to the maximum the minimum harvested energy power per energy vehicular receiver subject to the constraints in (13). For the baseline scheme 1, we adopt the zero-forcing approach to null out the undesired interference to the information vehicular receiver. In particular, we choose V and $V_{0}$ lying in the null space of the channel h. Furthermore, we employ the maximum ratio transmission for transmitting information to the information vehicular receiver. It can be observed that the proposed scheme closely approaches the performance of the perfect CSI scheme. On the other hand, it can be seen that the low computational complexity of the baseline scheme 1 comes at the expense of a significantly lower harvested energy power compared to the proposed scheme and the linear EH scheme. Indeed, the proposed scheme and the linear EH scheme fully use the CSI of all vehicular communication links and optimize the space spanned by the artificial noise and the energy signal for performing beamforming design. On the contrary, for the baseline scheme 1, the base station is unable to fully utilize the available degrees of freedom in beamforming design because the both the energy beam V and artificial noise $V_{0}$ are fixed. Finally, with the increase of *P*, the proposed scheme achieves higher harvested energy power than other schemes for every channel realization. The reason for this is that the proposed scheme takes into account the nonlinearity of practical EH circuits leading to mismatches in beamforming design.

**Figure 3:**
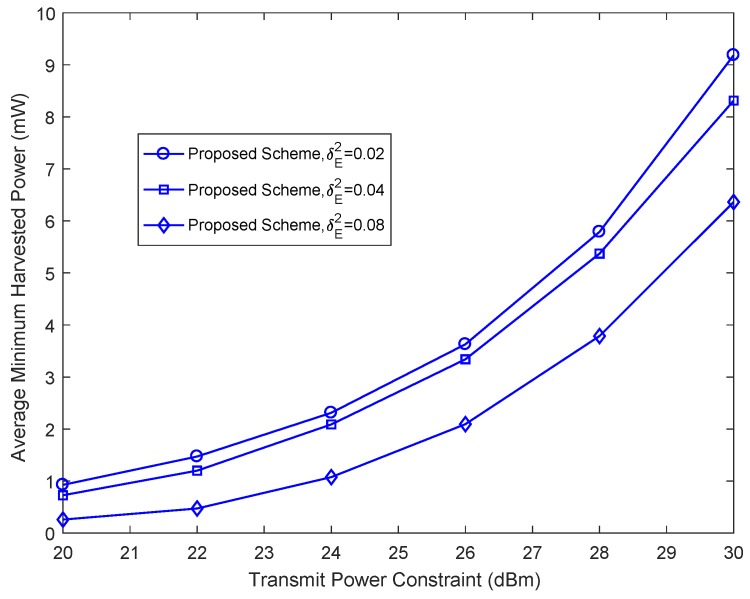
Average minimum harvested power per energy vehicular receiver versus total transmit power *P* with different $\delta_{E}^{2}$.

In Figure 3, we study the average minimum harvested energy power per energy vehicular receiver versus the maximum power of the base station, *P*, for $K_{E}=3$ energy vehicular receivers, and different than the maximum channel estimation errors, $\delta_{E}^{2}$. As can be seen, with the increase of *P*, the average minimum harvested energy power per energy vehicular receiver increases for increasing maximum channel estimation error, $\delta_{E}^{2}$. Indeed, with increasing the imperfectness of channel estimation, the base station has to allocate less power to the artificial noise and energy signals to ensure vehicular secure communication.

**Figure 4:**
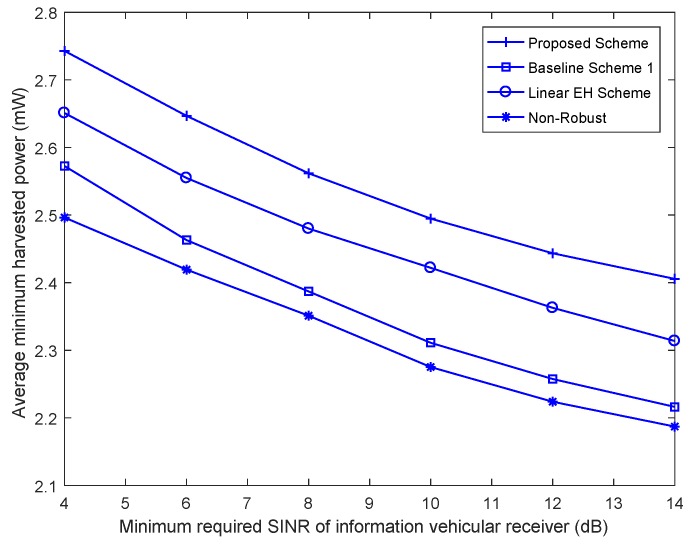
Average minimum harvested power per energy vehicular receiver versus the minimum required SINR of the information vehicular receiver, *r*.

In Figure 4, we move on to investigating how the average harvested energy power per energy vehicular scales with the minimum required SINR of the information vehicular receiver, *r*, for $K_{E}=3$ energy vehicular receivers, $\delta_{E}^{2}=0.02$, and different beamforming design schemes. It can seen that the average minimum harvested energy power per energy vehicular receiver of the proposed scheme is a monotonically increasing function of *r*. This is owing to the fact that there is a trade-off of the proposed beamforming design scheme between power collecting of the energy vehicular receivers and the guarantee of physical layer secure of information vehicular receiver. In particular, the achieved SINR of the information vehicular receiver and the minimum harvested energy power per energy vehicular receiver can not be maximized simultaneously with fixed $r_{k}$ and vice versa. Among all the considered beamforming design schemes, the proposed nonlinear EH scheme still achieves a better performance than the other beamforming design schemes.

**Figure 5:**
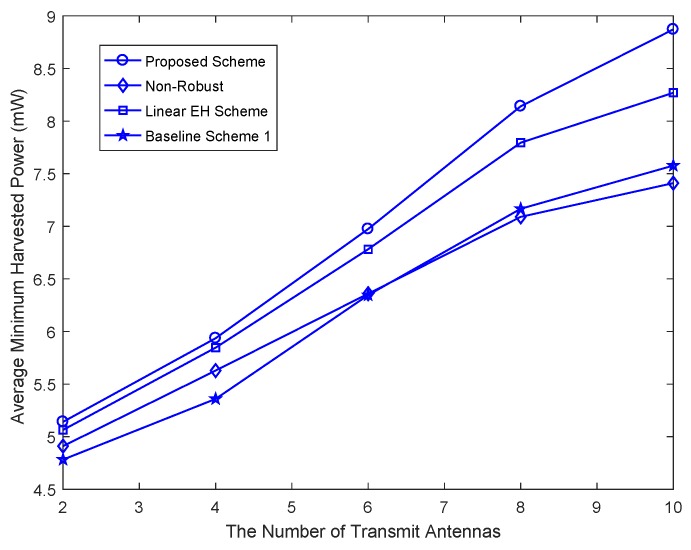
Average minimum harvested power per energy vehicular receiver versus the number of transmit antennae, $N_{T}$.

Figure 5 shows the average minimum harvested energy power per energy vehicular receiver versus the number of the transmit antenna, $N_{T}$, for $K_{E}=3$ energy vehicular receivers, $\delta_{E}^{2}=0.02$, and different beamforming design schemes. It can observed that the average minimum harvested energy power per energy vehicular receiver in the considered system increases with the increasing number of antennae. These results advise that a higher amount of energy is available in the RF for energy collecting when the number of the transmit antennae increases. This is owing to the fact that with more transmit antennae the direction of energy beamforming matrix V can be more accurately steered towards the energy vehicular receivers, which further increase the amount of energy available in the RF for energy collecting. Among all the considered beamforming design schemes, the proposed beamforming design scheme also achieves a better performance than the other beamforming design schemes.

**Figure 6:**
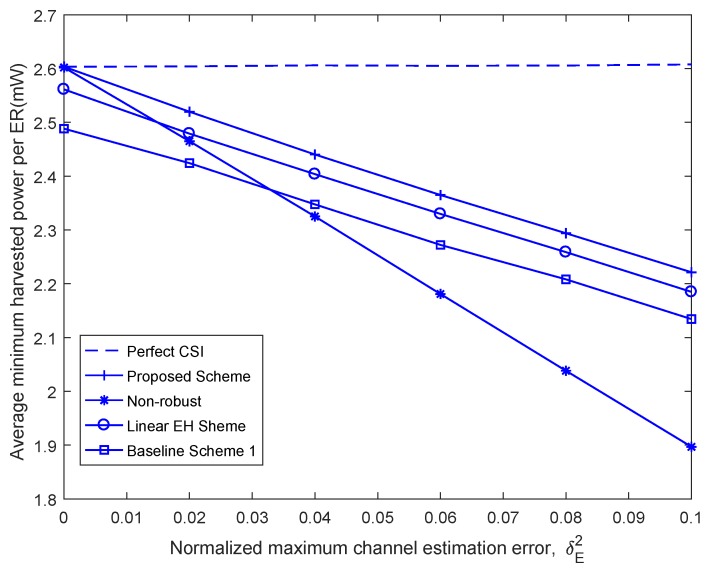
Average minimum harvested power per energy vehicular receiver versus channel estimation error, $\delta_{E}^{2}$.

Figure 6 illustrates the average minimum harvested energy power per energy vehicular receiver versus the maximum channel estimation error, i.e., $\delta_{E}^{2}$, for different beamforming design schemes. As seen in Figure 6, it can be seen that the minimum harvested energy per energy vehicular receiver of the proposed robust beamforming design scheme decreases with the increase of the estimated channel error, $\delta_{E}^{2}$, which confirms the motivation of the worse case robust optimization. Moreover, we learn intuitively that the proposed nonlinear EH beamforming design scheme performs considerably better than other beamforming design schemes but worse than the perfect case.

## 5. Conclusions

In this paper, we formulated the beamforming design for secure V2X communication systems with an RF energy collecting vehicular receiver as a non-convex optimization problem, which took into account the imperfection of CSI and the nonlinearity of practical EH circuits. The considered problem formulation supports the dual use of energy signals and artificial noise for efficient energy transfer and facilitating secure vehicular communication in the presence of the potential eavesdroppers (idle energy vehicular receivers). Owing to the intractability of the resulting max-min harvested energy power per energy vehicular receiver problem, the optimization problem was reformulated and solved with a semidefinite relaxation technique and variable substitutions. Numerical results showed the excellent performance of the proposed robust secure beamforming design algorithm.
